# Empowering brain tumor management: chimeric antigen receptor macrophage therapy

**DOI:** 10.7150/thno.98290

**Published:** 2024-09-03

**Authors:** Fan Feng, Jianyu Shen, Qichao Qi, Yulin Zhang, Shilei Ni

**Affiliations:** Department of Neurosurgery, Qilu Hospital and Institute of Brain and Brain-Inspired Science, Cheeloo College of Medicine, Shandong University, 107 Wenhua Xi Road, Jinan, 250012, Shandong, China.

**Keywords:** Brain tumor, Immunotherapy, Chimeric antigen receptor, Macrophage

## Abstract

Brain tumors pose formidable challenges in oncology due to the intricate biology and the scarcity of effective treatment modalities. The emergence of immunotherapy has opened new avenues for innovative therapeutic strategies. Chimeric antigen receptor, originally investigated in T cell-based therapy, has now expanded to encompass macrophages, presenting a compelling avenue for augmenting anti-tumor immune surveillance. This emerging frontier holds promise for advancing the repertoire of therapeutic options against brain tumors, offering potential breakthroughs in combating the formidable malignancies of the central nervous system. Tumor-associated macrophages constitute a substantial portion, ranging from 30% to 50%, of the tumor tissue and exhibit tumor-promoting phenotypes within the immune-compromised microenvironment. Constructing CAR-macrophages can effectively repolarize M2-type macrophages towards an M1-type phenotype, thereby eliciting potent anti-tumor effects. CAR-macrophages can recruit T cells to the brain tumor site, thereby orchestrating a remodeling of the immune niche to effectively inhibit tumor growth. In this review, we explore the potential limitations as well as strategies for optimizing CAR-M therapy, offering insights into the future direction of this innovative therapeutic approach.

## INTRODUCTION

Despite remarkable progress in the conventional treatment modalities for brain tumors (BTs), including surgical resection, radiotherapy, and systemic chemotherapy, patient outcomes remain unsatisfactory, primarily due to the intricate interplay within the BTs microenvironment 1, 2. The microenvironment, characterized by a complex network of immune cells, stromal elements, and cytokines, exerts profound influence on tumor progression and therapeutic responses. Central to this are tumor-associated macrophages (TAMs), a prominent constituent of the immune infiltrate within BTs 3. TAMs exhibit a remarkable plasticity and can adopt divergent functional states, often influenced by cues from the tumor microenvironment 4. In the central nervous system, TAMs promote tumor growth and metastasis by fostering an immunosuppressive microenvironment characterized by the secretion of factors such as transforming growth factor-β (TGF-β) and interleukin-10 (IL-10), which inhibit anti-tumor immune responses and promote angiogenesis 5. However, the balance between pro-tumorigenic and anti-tumorigenic functions of TAMs is delicately regulated by various factors, such as hypoxia, nutrient deprivation, and tumor-derived soluble factors 6.

Harnessing the immunomodulatory properties of TAMs represents a promising avenue for enhancing anti-tumor immune responses and improving patient outcomes 7. However, challenges such as TAM heterogeneity, plasticity, and resistance mechanisms need to be overcome to realize the full therapeutic potential of targeting TAMs in BTs therapy 8. In this regard, engineered macrophages equipped with Chimeric Antigen Receptors (CARs) tailored for tumor specificity emerge as a compelling strategy. These engineered macrophages possess a multifaceted arsenal of capabilities crucial for combating tumor progression within the intricate microenvironment of the BTs 9. By leveraging their inherent migratory ability and capacity for infiltrating deep into tumor sites, CAR-macrophages (CAR-Ms) can directly engage with tumor cells and mount potent immune responses 10, 11.

Facilitated by the innate ability to traverse the blood-brain barrier and penetrate deep within the tumor parenchyma, CAR-Ms navigate the complex landscape of the BTs microenvironment with precision 12, 13. Once deployed within this hostile terrain, CAR-Ms unleash a formidable immune response characterized by targeted phagocytosis of tumor cells and efficient presentation of tumor antigens 14. Concurrently, the presentation of tumor antigens by CAR-Ms facilitates the activation and proliferation of cytotoxic T cells, bolstering the adaptive immune response against the tumor 15. This dual immunomodulatory action holds significant promise for overcoming the immunosuppressive microenvironment characteristic of BTs and fostering durable anti-tumor immunity 16.

Compared to Chimeric Antigen Receptor T cell (CAR-T) therapy, CAR-M therapy demonstrates distinct advantages in terms of therapeutic efficacy and suitability for targeting BTs. CAR-Ms exhibit superior infiltration and activation of both innate and adaptive immune responses within the BTs microenvironment 17. This enhanced immunological engagement holds significant promise for overcoming the immunosuppressive barriers characteristic of BTs and bolstering anti-tumor immunity 18. As such, the burgeoning field of CAR-M therapy in BTs treatment merits heightened attention and exploration. Its potential to revolutionize immunotherapy by leveraging the unique attributes of macrophages to orchestrate robust anti-tumor immune responses represents a transformative paradigm shift in the quest for more effective treatments against this devastating disease 19.

## BIOLOGY OF BRAIN TUMOR AND MARCROPHAGES

### Biology and Microenvironment of Brain Tumor

Primary BTs originate within the brain and are further categorized based on their cellular origin and molecular characteristics, such as gliomas and medulloblastomas. Secondary BTs originate from cancer cells that have disseminated from other regions of the body which are invariably malignant and are categorized according to the primary site of origin 20. BTs exhibit diverse structural characteristics depending on their type and grade. High-grade tumors, such as glioblastoma multiforme (GBM), are characterized by rapid growth, necrosis, and extensive vascularization 21. Low-grade tumors, such as grade I astrocytoma, tend to grow slowly and have well-defined borders 22. The structural complexity of BTs poses challenges for surgical resection and treatment. The treatment of BTs is a multifaceted process that depends on the specific characteristics of the tumor, including type, size and location 23. Surgical resection remains the primary treatment modality, with the objective of removing as much of the tumor as possible. Adjuvant therapies, including radiation and chemotherapy, are employed to eliminate residual tumor cells 24. Even with all the continued advances in drug discovery and formulation that have substantially improved outcomes in systemic cancers, there has been disappointingly little impact on tumors of the brain 9. The advent of targeted therapies and immunotherapies, such as CAR cell therapy, has demonstrated potential in treating specific tumor subtypes by targeting tumor-specific antigens. Nevertheless, challenges such as tumor heterogeneity and the intricate immune microenvironment continued research to optimize treatment outcomes 25.

The immune microenvironment of BTs is characterized by a complex interplay of immune cells, stromal cells, and tumor cells, contributing to an immunosuppressive milieu that supports tumor progression. Key players in this environment include TAMs, microglia, and regulatory T cells, which collectively promote immune evasion and tumor growth 26. Although expected to induce apoptosis of tumor, cytotoxic T cells and natural killer (NK) cells are suppressed within TME either by direct contact with tumor or under the influence of inhibitory factors contributed by Treg cells and TAMs 27, 28. TAMs also help in the promotion of tumors angiogenesis and proliferation. The structurally and functionally aberrant tumor vasculature contributes to the pro-tumorigenic and immunosuppressive TME by maintaining hypoxia, acidosis, and high interstitial pressure, while simultaneously generating a physical barrier to T cell infiltration 29. Of note, hypoxia and hyperlactatemia induce metabolic reprogramming and epigenetic changes which are interconnected, and to a large extent, metabolic state dictated epigenetics in BTs. Furthermore, epigenetic alterations are associated with all aspects of BTs, from BTs initiation to cancer progression and metastasis 30, 31.

### The Role of Macrophages in Brain Tumor

Macrophages are innate immune cells that participate in immune defense, tissue homeostasis, and the regulation of diseases. TAMs are defined as macrophages that infiltrate tumor, which are an integral part of the immunity, exerting significant influence on the development and treatment of BTs 32. TAMs play a dual role in tumor biology, either participating in tumor surveillance and eradication or facilitating tumor progression 33, 34. TAMs polarize into the M1-like phenotype upon exposure to interleukin-12 (IL-12), tumor necrosis factor-α (TNF-α), interferon-γ (IFN-γ), granulocyte-macrophage colony-stimulating factor (GM-CSF), and bacterial lipopolysaccharide (LPS), characterized by CD68, CD80, CD86, major histocompatibility complex II (MHCII), and inducible nitric oxide synthase (iNOS) expression, exhibiting anti-tumor activity, reactive oxygen species (ROS) secreting, enhanced antigen presentation, proinflammatory cytokine production, and involvement in T helper (Th) type 1 responses 35, 36. Exposure to IL-4, 5, 10, 13, colony-stimulating factor 1 (CSF1), transforming growth factor-beta (TGF-β), and prostaglandin E_2_ (PGE_2_), TAMs polarize into the M2-like phenotype, characterized by CD206, CD204, vascular endothelial growth factor (VEGF), CD163, and arginase-1 (Arg-1) expression, supporting protumor functions and contributes to BTs proliferation, growth, and invasive metastasis by promoting angiogenesis and stemness, creating an immunosuppressive environment that facilitates immune escape, thereby enhancing drug resistance and influencing neuronal activity 37-39.

Macrophages are critical effectors of antibody-based cancer therapy, and as antigen-presenting cells, activated macrophages can play a critical role in promoting an adaptive anti-tumor immune response. These considerations spurred previous attempts to transfer autologous macrophages to patients with tumors but failed to demonstrate notable anti-tumor efficacy 40, 41.

### CAR-Macrophages: What They Are and How They Work

**Figure 1:**
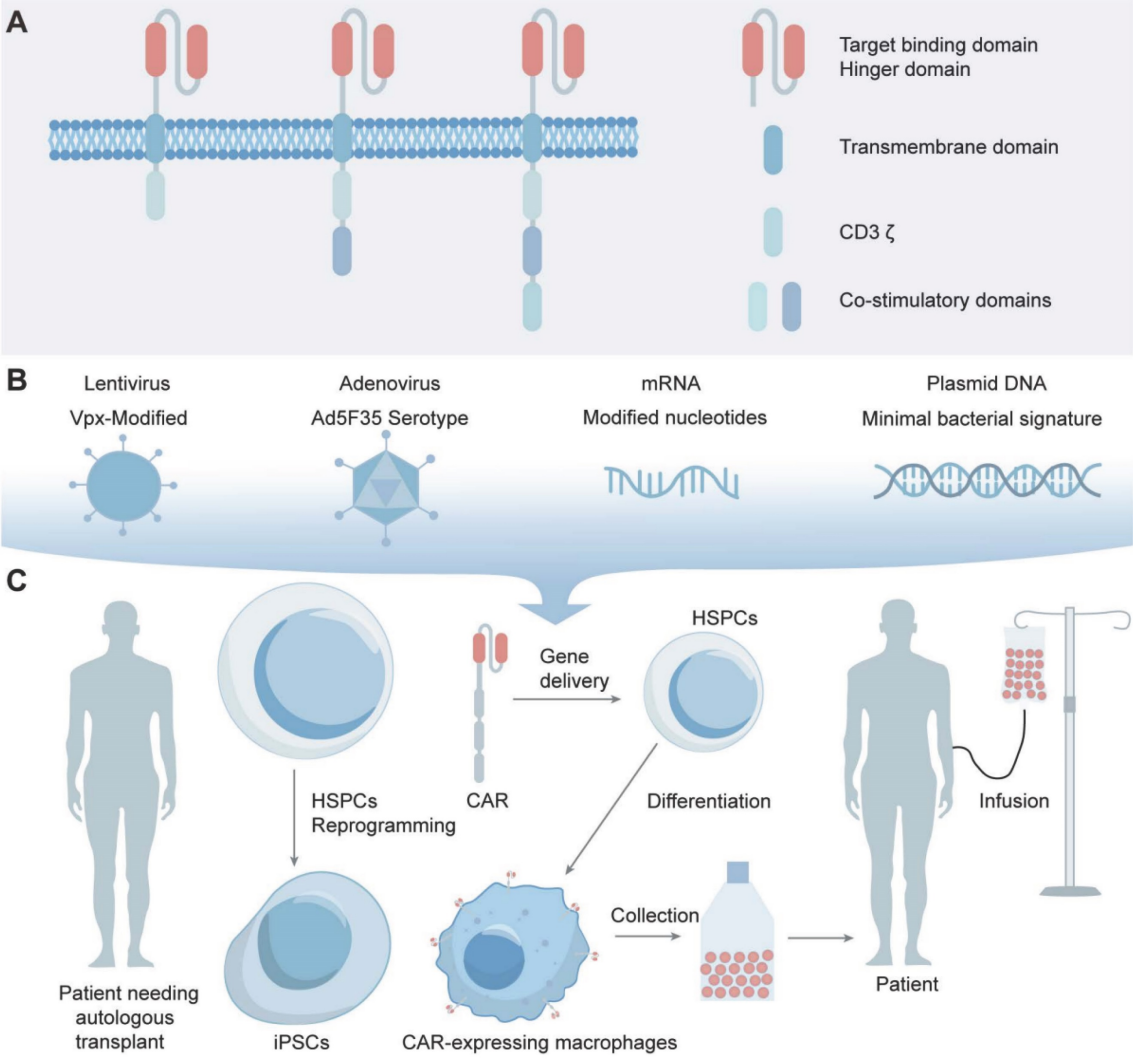
The structure of CARs and the construction of CAR-Macrophages. (**A**) CAR-macrophages possess extracellular antigen-binding domains, hinge regions, transmembrane domains and intracellular domains. (**B, C**) Advances in macrophage transduction technology have made it possible to transfer CARs into macrophages.

**Figure 2:**
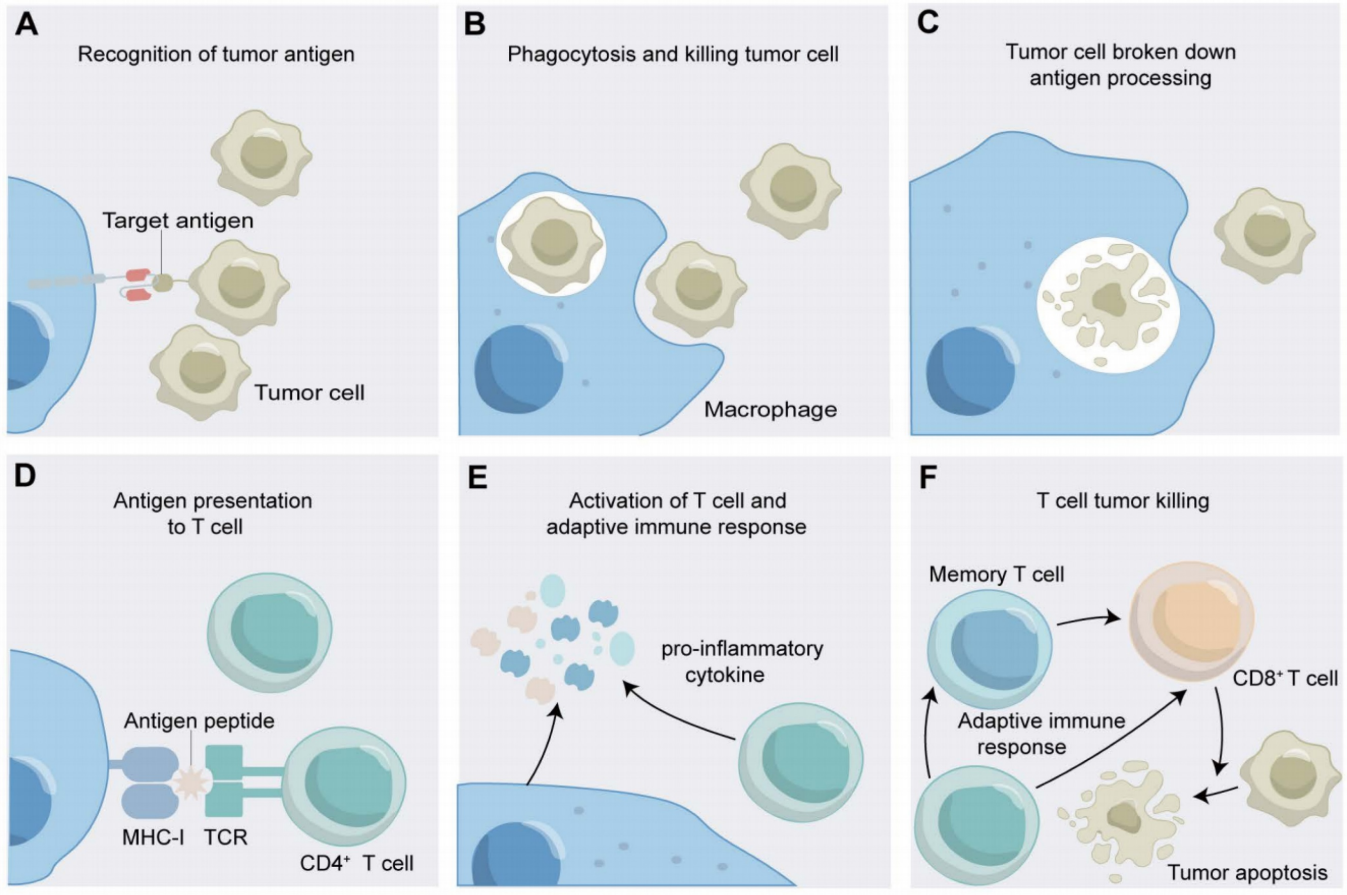
The Mechanism of Action of CAR- CAR-Macrophages. (**A**) CAR-macrophages recognize tumor antigens; (**B**) CAR-macrophages phagocytose tumor cells; (**C**) CAR-macrophages breakdown tumor antigens; (**D**) CAR-macrophages present tumor antigens to T cells; (**E**) CAR-macrophages activate adaptive immune response. (**F**) The adaptive immune response kills tumor cell.

As an advancement, through receptor engineering, CAR-Ms possess extracellular antigen-binding domains, hinge regions, transmembrane domains and intracellular domains (Figure 1A) 42. An antigen-binding domain is usually single-chain Fv (scFv) with a simple ectodomain and more exotic recognition components, yet recent innovations have expanded the constitution to include elements from other domains such as nanobodies, designed ankyrin repeat proteins (DARPins), ligands, or receptors 43. The hinge region, an extracellular structure, links the antigen-binding domain (ABD) to the transmembrane domain, where variations in composition or length can influence CAR-antigen binding and signaling 44. The transmembrane domain, characterized by a hydrophobic alpha helix that spans the cell membrane, anchors the CARs in the membrane, affecting its expression, stability, and function 45. The intracellular domain is the functional end of the receptor. In CAR-Ms, the intracellular domains are typically derived from signaling molecules that can induce macrophage activation and polarization 17. The most commonly used intracellular domain is CD3ζ chain, which is derived from the T-cell receptor complex and is responsible for initiating the primary activation signal 46. However, to enhance the antitumor efficacy of CAR-Ms, additional costimulatory domains are often incorporated 47. After receptor engineering, once the extracellular antigen-binding domain recognizes the antigen, intracellular signal transduction is initiated. CAR-Ms directly interact with the kinase Syk, which contains tSH2 domains, and utilize the intracellular CD3ζ domain, which contains immunoreceptor tyrosine-based activation motifs (ITAMs), to transmit phagocytic signals 48, 49. In addition to CD3ζ, FcRγ effector function is antibody-dependent cellular phagocytosis (ADCP), while Megf10 can also induce the phagocytic function. All of these possess intracellular domains containing ITAMs 50. After CAR-Ms identify and phagocytose tumor cells via CAR receptors, tumor engulfment is initiated in CAR-Ms. The subsequent degradation of tumor antigens leads to their presentation to CD4^+^ T cells through MHC molecules, which activate the adaptive immune response to effectively eliminate tumor cells (Figure 2) 47.

## DEVELOPMENT OF CAR-MARCROPHAGES FOR BRAIN TUMOR TREATMENT

### Historical Context and Early Experiments with CAR-T cells

**Figure 3:**
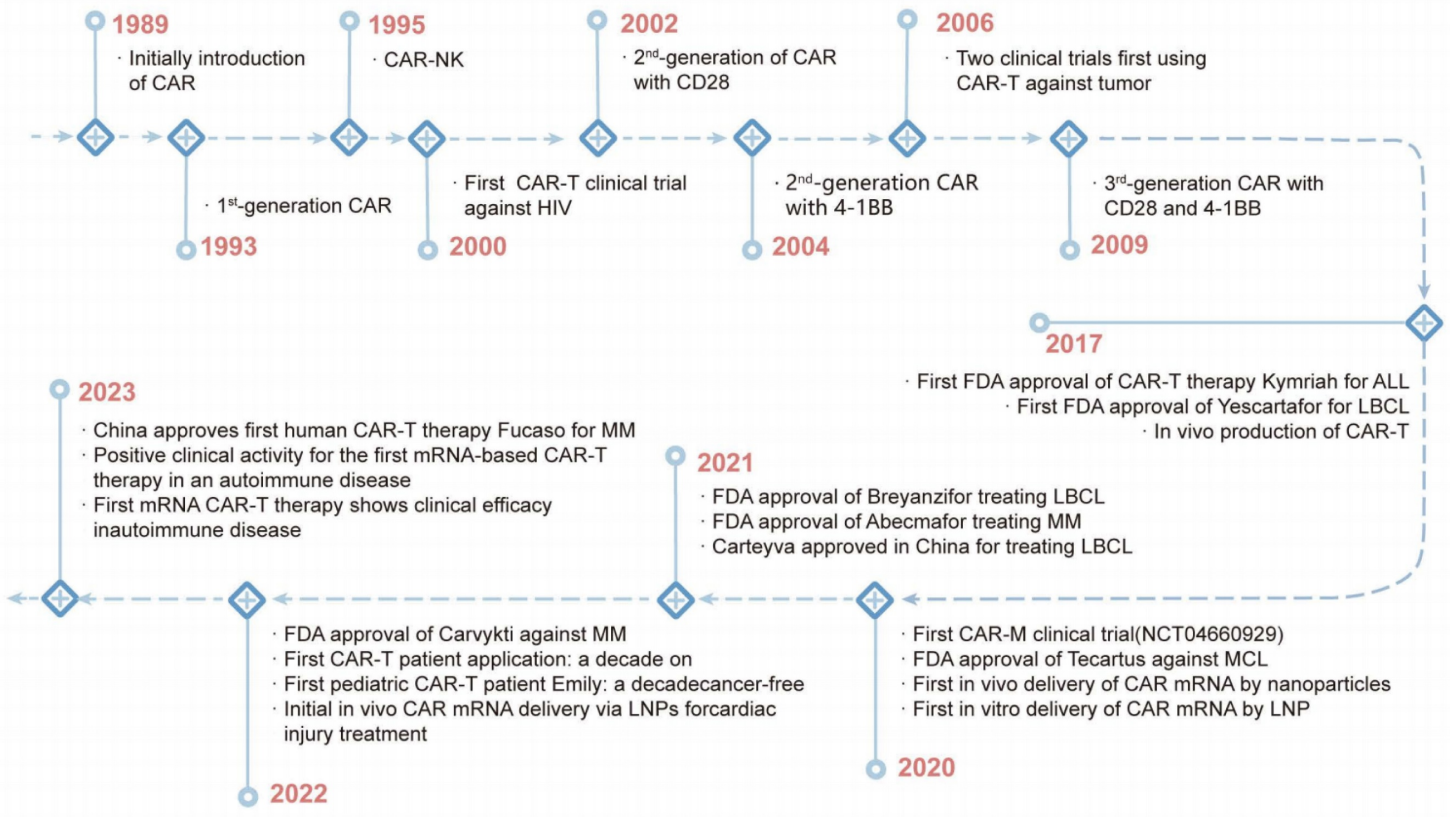
The History of CAR Cell Therapy Development.

CAR technology was first described by Kuwana, Y. in 1987. This was followed by the discovery of the intracellular signaling domain of CD3ζ, which activates T-cell signaling 51. The discovery enabled the development of first-generation CARs combining scFv domains, CD4 and other components in 1991 52. However, clinical trials using CAR-T therapy to eradicate HIV-infected cells and solid tumors were unsuccessful in the mid-1990s 52, 53. In 2003, a study showed that human CD19-directed CAR-T cells could kill leukemia cells in a mouse model, and CAR-T therapy has become a popular treatment for leukemia 54. In 2010, the FDA approved the first clinical trials of CD19 CAR-T cells, and then two more products (Kymriah and Yescarta) were approved by the FDA in 2017 (Figure 3) 53. To date, six CAR-T therapies have received FDA approval for hematological malignancies: Kymriah, Yescarta, Tecartus, Breyanzi, Abecma, and Carvykti 55.

### Advantages and Limitations of CAR-Macrophages Compared to CAR-T Cells

CAR-M therapy and CAR-T therapy both utilize CARs to target and eliminate cancer cells, but their mechanisms of action differ significantly. CAR-T cells, derived from T lymphocytes, primarily exert their anti-tumor effects through cytotoxicity. Upon binding to the target antigen, CAR-T cells are activated through the CD3ζ signaling domain, releasing perforin and granzymes to induce apoptosis in target cells 56. Despite the potential of T cells targeted to solid tumors, their efficacy is hampered by the TME, which impairs their infiltration and cytotoxic capabilities 57, 58. In addition, T-cell therapy may induce severe side effects such as Graft-Versus-Host Disease (GVHD), Cytokine Release Syndrome (CRS), “on-target/off-tumor” toxicity, and immune effector cell-associated neurotoxicity syndrome (ICANS) 59. Another challenge lies in the scarcity of tumor-specific antigens in solid tumors amenable to CARs, thereby limiting the effectiveness of CARs 60. Furthermore, the high cost and time-consuming production of CAR-T therapy hinder widespread accessibility.

In contrast, CAR-Ms are engineered macrophages that express CARs similar to those in CAR-T cells 61. However, the intracellular domains are often modified to include signaling motifs that promote macrophage activation and polarization. CAR-Ms, derived from macrophages, utilize phagocytosis to engulf and digest cancer cells 15. Additionally, CAR-Ms secrete pro-inflammatory cytokines that modulate the tumor microenvironment and recruit adaptive immune cells, enhancing the overall anti-tumor response 25. CAR-Ms have the potential to be an effective treatment for solid tumors due to their strong infiltration capacity and ability to act in both antigen-dependent and antigen-independent ways. The plasticity of macrophages allows for reversion to the M1-like phenotype characterized by antigen presentation to activate the adaptive immune response, further remodeling the matrix in the TME and recruiting more effector lymphocytes 48. Furthermore, CAR-Ms are long-lived, have the ability to induce cytotoxic activity in the tumor niche and can be chemically modified to enhance their drug delivery 62-66, which means they can provide long-term therapeutic effects without the risk of toxicity associated with T-cell therapies, such as CRS and neurotoxicity 67.

### Techniques for Developing CAR-Macrophages for Cancer Immunotherapy

CAR-Ms, while exhibiting potential in solid tumor therapeutics, encounter a multitude of formidable challenges 11. Leveraging CAR-T therapy advancements, progress in optimizing CAR-Ms has been made, focusing on transfection efficiency and safety.

The main challenge of CAR-M therapy is the efficient transduction of the CAR gene or plasmid into macrophages. Macrophages possess a natural environment that is resistant to viral replication, as well as the ability to recognize and respond to foreign nucleic acids, exhibiting a high resistance to gene engineering 68. Advances in macrophage transduction enable the transfer of CARs into macrophages, allowing for the engineering of cells to express and secrete therapeutic proteins targeting BTs. CD46, highly expressed in monocytes and macrophages, serves as the receptor for B-group adenoviruses such as Ad35, facilitating viral entry 69, 70. To improve transduction efficiency, Vpx, a viral accessory protein that mediates SAMHD1 degradation, was incorporated into the lentivirus at the packaging stage to overcome SAMND1 inhibition and enabling viral transduction 71. Ad5f35, a replication-incompetent chimeric adenoviral vector, maintains transduction of macrophages persists for at least one month *in vitro* and at least 62 days* in vivo*, indicating its potential as a vector for the long-term delivery of gene payloads to myeloid cells (Figure 1B) 48. The vector not only efficiently delivers CAR genes to macrophages, but also activates the macrophage inflammasome, providing a proinflammatory stimulus that synergizes with CAR activity. Despite the advances, the clinical application of adenoviral vectors like Ad5f35 is limited due to concerns over oncogenic potential and immune reactions 72, 73.

**Figure 4:**
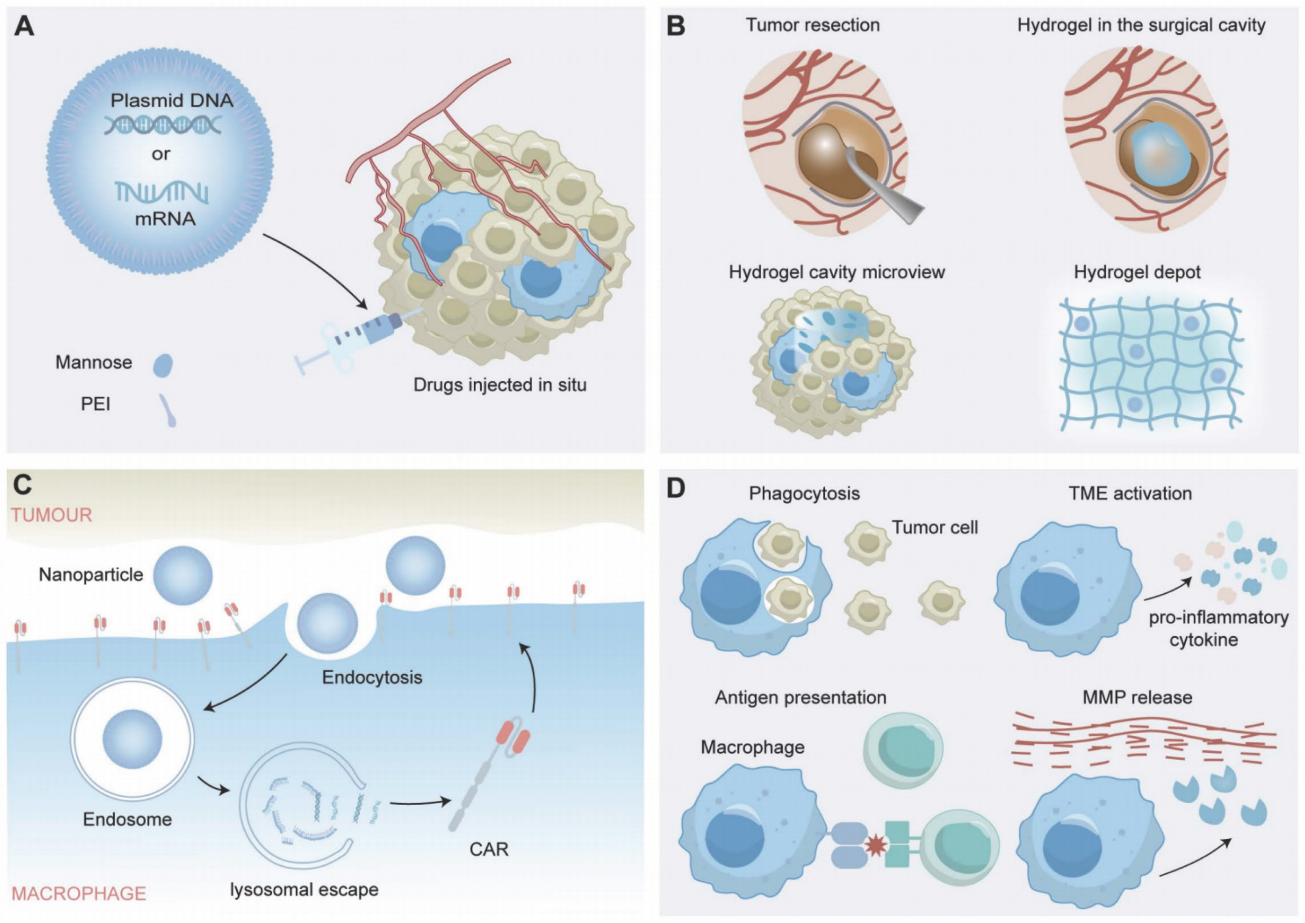
The Application of CAR-Macrophage Therapy in Postoperative Tumors. (**A, B, C**) The applications of nanotechnology, gene delivery, and synthetic biology in CAR-Macrophages. (**D**) The immune regulatory role of CAR-macrophages in the tumor microenvironment.

Recent studies into non-viral methods have shown promise as a solution for gene therapy delivery challenges, merging the fields of nanotechnology, gene delivery, and synthetic biology to potentially transform cancer immunotherapy and immunomodulation strategies 74. Recent studies highlight the potential of nanocomplexes to address CAR-M therapy challenges, such as reducing costs, simplifying manufacturing processes, and decreasing tumorigenic risks associated with viral vector transduction 75. The nanocomplexes injected *in vivo* contain mannose-conjugated polyethyleneimine (MPEI), which significantly enhanced the macrophage targeting efficiency both *in vitro* and *in vivo* as mannose receptors are overexpressed in macrophages, and CAR-IFN-γ-encoding plasmid DNA with a nonviral piggyBac transposon system 76, which is a mobile genetic element that can be efficiently transposed between vectors and chromosomes via a "cut and paste" mechanism for the sustained expression of CAR transgenes 77. The nanocomplexes induced a shift in macrophages from the M2-like to the M1-like phenotype and maintained the anti-tumor state post-phagocytosis of malignant cells *in vitro*. They also halted tumor growth in Neuro-2a tumor-bearing mice without causing systemic toxicity, through a reduction in Treg cells and an increase in activated CD8+ T cells, facilitated by CAR-dependent tumor cell phagocytosis (Figure 4A) 78. Recent studies have utilized a CpG-free plasmid, which avoids Toll-like receptor 9 (TLR9) recognition, inflammatory responses and transgene silencing, is effective for delivering a small plasmid (pCGfd-GFP) encoded by the vector. The method showed high transfection efficiency, sustained transgene expression and good cell viability in the transfections of Raw 264.7 and primary bone marrow-derived macrophages. Moreover, the method engineers the macrophages secreting anti-EGFR scFv-Fc, capable of effectively targeting and phagocytizing EGFR-expressing tumor cells through ADCP 65, 79.

Based on these innovative approaches of gene delivery and macrophage-targeted therapies, it is critical to further explore cutting-edge strategies that harness the potential of synthetic biology to augment macrophage functions within the complex TME. Researchers have demonstrated that a cavity-injectable nanoporter-hydrogel superstructure, which generates glioblastoma stem cells (GSCs)-specific CAR-Ms around the cavity, is effective in preventing BTs recurrence 80. A nuclear localization sequence (NLS) peptide-synthesized nanomicelle was utilized for nuclear-targeted CAR plasmid (pCar) gene delivery and further coated with citraconic anhydride-modified dextran (CA-dextran) to achieve CD206-targeted pCAR-laden nanomicelles 80. Brain ECM-derived laminin peptide and an immunostimulatory peptide were used as the precursors of the hydrogel. After intracavity delivery, the CAR gene-laden nanoporter introduced GSC-targeted CAR genes into macrophage nuclei, thus generating CAR-Ms in mouse models of BTs (Figure 4B) 80.

The widespread use of CRISPR/Cas9 in cell immunotherapy has yielded significant outcomes, particularly in CAR-Ms, where it effectively facilitates CAR gene knock-in and enhances macrophage proinflammatory effector functions to counter the TME 81. CRISPR/Cas9 genome editing involves the following three steps: recognition, cleavage, and repair. An sgRNA is designed to recognize the target gene sequence through complementary base pairing. The Cas9 protein is then used to cleave DNA at the site, and the cell repair machinery repairs the break either through nonhomologous end joining or homology-directed repair 82. The CRISPR/Cas9 complex can be delivered as plasmid DNA (pDNA), messenger RNA (mRNA), or ribonucleoprotein (RNP), with direct RNP delivery being advantageous due to its avoidance of many pitfalls associated with pDNA or mRNA delivery. RNP delivery facilitates the swiftest genome editing by eliminating the need for intracellular transcription and translation 80, 83-85. In a study, CRISPR/Cas9-mediated depletion of SIRPα, a “don't eat me” signal of SIRPα/CD47, demonstrated synergistic efficacy in anti-tumor responses, whereas SIRPα knockout alone failed to enhance anti-tumor macrophage responses 86. Additionally, researchers utilized CRISPR/Cas9 to integrate the anti-GD2 CAR into AAVS1 locus of hPSCs, resulting in CAR-Ms effectively targeting and eliminating GD2-expressing neuroblastoma and melanoma *in vitro* and *in vivo* 87. Furthermore, CRISPR/Cas9 technology enhances CAR-Ms function by targeting regulators such as the aconitate decarboxylase 1 (ACOD1) and Kelch-like ECH-associated protein 1 (KEAP1), leading to improved persistence, polarization, ROS production, phagocytosis, and cytotoxicity *in vitro*, ultimately exhibiting high antitumor efficacy in mouse models of ovarian and pancreatic cancer. Moreover, synergistic effects were observed when combining this approach with immune checkpoint inhibitors 88. Alternatively, catalytically dead Cas9 (dCas9) fused to transcriptional repression domains, 89 such as KRAB domains, chromatin remodeling factors, 90 and histone methylases, can be utilized to epigenetically silence targets known to precipitate M2-like phenotype polarization 91.

Selecting optimal cell sources is a pivotal consideration in cell therapy 92. Peripheral blood mononuclear cells (PBMCs) offer easy access and are enriched with various immune cells for immunotherapy, although they exhibit limited genetic manipulation rates 17. Alternatively, cell lines like THP-1, which possess macrophage-like characteristics induced by substances such as phorbol-12-myristate-13-acetate (PMA) and macrophage colony-stimulating factor (M-CSF), present advantages in terms of homogenous genetic background, ease of culture, rapid proliferation, and higher safety compared to PBMC-derived macrophages 93. However, THP-1 lacks certain features such as LPS tolerance and specific cytokine secretion patterns, making them a viable but distinct option from PBMCs 94. Induced pluripotent stem cells (iPSCs), reprogrammed from PBMCs, can differentiate into various somatic cell types, including immune cells. Rapid progress has been made in differentiating iPSCs into immune cells, including CAR-T and CAR-NK cells, demonstrating excellent cytotoxic effects against tumors. Researchers have developed iPSC-derived, CAR-expressing macrophages (CAR-iMac) for use in cancer immunotherapy 95, 96. Based on the characteristics of iPSCs, researchers have obtained CAR-iMac with high yield and purity, exhibiting M1-associated gene expression and possessing phagocytic function. Cocultured with lymphoma cells expressing CD19 or ovarian cancer cells expressing the mesothelin, CAR-iMac were cytotoxic, employed ADCP, and polarized toward an M1-like phenotype (Figure 1C) 97.

The first-generation CAR-Ms were designed by incorporating the CD3ζ activating domain, similar to the first-generation CAR-T cells. This design aimed to leverage the phagocytic abilities of macrophages to target and eliminate tumor cells 9, 25. It is not feasible to polarize macrophages towards a durable M1-like pro-inflammatory state in theory. Therefore, the design of a new macrophage-specific CAR to confer CAR-Ms with both phagocytosis abilities and polarization functions would contribute to fight against BTs 98. Researchers engineered induced pluripotent stem cell-derived macrophages (iMACs) with toll-like receptor 4 intracellular toll/IL-1R (TIR) domain-containing CARs 99. The design of a tandem CD3ζ-TIR dual signaling CAR endows iMACs with both target engulfment capacity and antigen-dependent M1 polarization and M2 resistance in a nuclear factor kappa B (NF-κB)-dependent manner, as well as the capacity to modulate the tumor microenvironment. The second-generation CAR-iMACs demonstrated superior antitumor functions, including orthogonal phagocytosis and polarization, resulting in a markedly enhanced antitumor effect over first-generation CAR-Ms 9, 99, 100.

### Clinical Studies of CAR-M Therapy

The accumulating evidence attesting to the efficacy, security, and feasibility of CAR-M therapy is intensifying the enthusiasm for launching a clinical trial of this treatment. Despite the safety and efficacy of CAR-M therapy has been validated in animal experiments, clinical trials are crucial for assessing the safety and efficacy 101. To date, a few clinical trials of CAR-M therapy are conducted and registered on clinicaltrials.gov. Currently, only three clinical trials associated with CAR-M therapy are in progress, and two clinical studies of CAR-monocyte therapy are also underway.

CT-0508 was developed by Carisma Therapeutics, the first CAR-M approved for clinical trials (NCT04660929). CT-0508 utilized Ad5f35-transduced PBMC-Macs to express HER2-targeted CARs and was administrated to 18 patients with relapsed or refractory tumors overexpressing HER2. Preliminary data reveals that CT-0508 is generally safe and well-tolerated with no dose-related toxicities. In addition to monotherapy, the combined treatment of CT-0508 and Pembrolizumab has been examined for the possible supplementary impacts in the context of the clinical trial 102. However, given the decision to prioritize CT-0525 as the product candidate in its HER2 program, Carisma has ceased further development of CT-0508 and new patients will no longer be enrolled in the Phase 1 clinical trial.

MAC-001, a CAR-M therapy developed by Macera Therapeutics, obtained approval to commence a phase l exploratory clinical trial targeting patients with advanced HER2-positive gastric cancer (NCT06224738). MAC-001 used an adenoviral vector system to genetically engineer autologous PBMC-Macs to express CAR molecules targeting HER2-positive tumors. Currently, the safety and feasibility of MAC-001 are being assessed in the ongoing clinical trial. Another clinical trial of CAR-M was an observational cohort study to determine the antitumor activity of CAR-Ms in breast cancer patients' derived organoids at different clinical stages. (NCT05007379).

Monocytes are precursor cells of macrophages with *in vivo* persistence and are also capable of differentiating into pro-inflammatory CAR-Ms with multimodal anti-tumor mechanisms of action. As research into CAR-Ms progresses, it is becoming increasingly evident that monocytes have the potential to play a significant role in CAR-M therapy.

Following the cessation of CT-0508 development, Carisma Therapeutics redirected the efforts towards CT-0525, an *ex vivo* gene-modified autologous CAR-Monocyte cellular therapy intended to treat solid tumors that overexpress HER2 metastasis. The Phase 1 study for CT-0525 is designed to assess the safety, tolerability, and the manufacturing feasibility of CT-0525, expected to conclude in Match 2026 (NCT06254807).

MCY-M11 developed by MaxCyte, in which mRNA transduction is performed in PBMCs to express CAR-targeted mesothelin, is designed to treat recurrent/refractory ovarian cancer and peritoneal mesothelioma patients (NCT03608618). The production of MCY-M11 exploited MaxCyte's Flow Electroporation® technology bypassing viral components or cell expansion. Promising results from single-round administrations of MCY-M11 are motivating, however, the final results have not been published yet 103.

MT-101 is an mRNA-engineered CAR-M therapy developed by Myeloid Therapeutics to treat patients with relapsed or refractory, CD5-positive peripheral T-cell lymphoma (PTCL). The safety, tolerability, and efficacy of MT-101 are being assessed in the ongoing Phase 1/2 clinical trial, expected to conclude in October 2024 (NCT05138458).

In contrast to the majority of existing CAR-M therapies, which are derived from fully developed immune cells, CAR-iMAC is generated from induced pluripotent stem cells that have been modified with CAR molecules and subsequently transformed into specialized macrophages (iPSCs) 97. SY001, an iPSC-derived CAR-Ms therapy developed by Cell Origin, is undergoing a single center, single-arm, dose-escalation, exploratory clinical trial to examine the safety, tolerability, pharmacokinetics and preliminary efficacy of SY001 from Cell Origin Biotechnology in patients with advanced solid tumors 104.

## MECHANISMS OF ACTION OF CAR MARCROPHEGES IN BRAIN TUMOR TREATMENT

### Target Antigens and Specificity of CAR-M Therapy

In BTs treatment, numerous tumor antigens have been identified and studied for the potential utilization in CAR-based immunotherapies. Here, we discuss the targets that have been explored in CAR-M therapy.

#### Erb-b2 Receptor Tyrosine Kinase 2 (HER2)

HER2 is a promising target for the treatment of BTs as the high abundance in BTs and low expression in brain tissues 105. In a study of CAR-T cell therapy on 16 patients with progressive GBM (NCT01109095), the safety of HER2-targeted CAR cell therapy was instituted. Though the clinical trial demonstrated an acceptable safety profile, the therapeutic efficacy fell short of the desired level of efficacy, highlighting the need for further improvements in enhancing the functionality, persistence, and expansion capabilities.

To take advantage of macrophages, CAR-Ms have been developed to specifically target HER2 and have been tested in preclinical models. CAR-M therapy had efficient tumor cell killing capacity and improved survival rates 48. To date, several CAR-Ms or CAR-monocytes targeting HER2 has been developed to treat HER2-positive solid tumors which are currently in the recruitment phase of clinical trials.

#### Epidermal Growth Factor Receptor (EGFR)

EGFR is one of the most commonly-mutated oncogenic sites in IDH-WT GBM. Approximately 50% of GBM multiforme samples exhibit mutations in their EGFR gene, with EGFRvIII (deletion of exons) being the most common mutation 106. As a potential marker for therapeutic treatment, several EGFR-targeted therapies have been investigated, including small molecule inhibitors such as gefitinib and dacomitinib, as well as antibodies, vaccines, CAR-T, and other approaches limit the number of EGFRs 107-113. However, these inhibitors have not been successful in patients suffering BTs with EGFR amplification (NCT01520870, NCT02447419), 107, 108 possibly due to the blood‒brain barrier. Therefore, researchers have proposed another potential therapeutic strategy utilizing CAR-Ms, which have high infiltration in BTs, that focuses on targeting EGFRvIII to suppress the growth of GBM and improve treatment efficacy.

#### Interleukin-13 Receptor Alpha 2 (IL-13Rα2)

IL-13Rα2 is specifically overexpressed in the majority of BTs (>60%) with a significantly lower expression observed in brain tissue 114-116. Clinically, IL-13Rα2 expression has been closely associated with the mesenchymal subtype of GBM, which has been identified as a prognostic indicator of lower patient survival rates 117. The therapeutic efficacy of IL-13Rα2 as a therapeutic target for BTs has been demonstrated in clinical trials 118, 119. A study by Brown and colleagues demonstrated the potential of multidose treatment with IL-13Rα2-CAR T cells, which induced complete tumor regression in 8 months in a patient with disseminated GBM (NCT02208362) 119. These findings about IL-13Rα2 indicate that it could be a promising target of CAR-M therapy for BTs.

#### Mesothelin (MSLN)

MSLN is named for the expression in mesothelin cells, overexpressed in a variety of malignancies including lung adenocarcinomas and some other squamous carcinomas 120, 121. CAR-T therapy targeting MSLN has been successively developed and applied showing great tumor elimination ability against cancers that highly express MSL 122, 123. Phase 1 clinical trial is now underway to evaluate the safety and feasibility of CAR-M therapy for targeting MSLN (NCT03608618). MSLN also represents a promising potential target for the treatment of brain metastases.

#### Others

Human B7-H3, encoded by *CD276* is overexpressed in many types of cancers, including GBM, and has been associated with tumor aggressiveness and poor prognosis 124-127. B7-H3 can act as an immune checkpoint that promotes tumor immune escape 128, 129. B7-H3 is not only highly expressed in most types of solid tumors, but is also present in the vessels and fibroblasts within tumors, 130 implying that CAR-Ms directed against B7-H3 may be able to eliminate tumor cells through direct targeting, disrupt the stroma, and even inhibit angiogenesis. The findings suggest that B7-H3 could be a promising target of CAR-M therapy for BTs.

Glypican-1 (GPC-1) is notably overexpressed in GBM, contrasting its low expression in healthy tissues including liver, pancreas, cervix, esophagus, and brain 131-135. GPC-1 is pivotal in regulating pathways related to tumor growth, angiogenesis, and invasion, highlighting its potential as a surface biomarker for developing targeted therapeutic and diagnostic agents 136.

Disialoganglioside 2 (GD2) has been identified as a marker of many malignancies and is specifically overexpressed in BTs, especially CSCs 137, 138. Animal models and clinical trials have confirmed the potential of CAR T cell therapy targeting GD2 as safe treatments for GBM 137, 139, 140. These findings indicate that GD2 is a potential therapeutic target for CAR-M therapy in BTs.

Prominin-1 (CD133) is a marker of CSCs in several human cancers, including GBM 141. Disabling the CD133 subpopulation, regardless of the expression level, has been shown to inhibit tumor growth and provide a survival benefit for preclinical models 142. Recently, investigators achieved successful *in situ* transfection of CARs targeting GBM CD133 into TAMs, leading to enhanced M1-like polarization and the inhibition of postoperative GBM recurrence. These findings suggest that CAR-Ms targeting for CD133 may hold promise as a potential treatment for BTs 80.

Although only a few of the potential targets for CAR-M therapy in BTs have been scientifically validated, their specific expression profile in BTs and application in CAR-T therapies demonstrates their potential to be exploited for CAR-M therapy in terms of BTs.

### Immunomodulatory Effects of CAR-Macrophages in the Brain Tumor Microenvironment

CAR-Ms are genetically engineered immune cells that express CARs on their surface, which allows them to specifically recognize and target tumor cells. In the context of the tumor microenvironment, CAR-Ms can exert various immunomodulatory effects, including the following (Figure 4D):

**Phagocytosis of Tumor Cells:** CAR-Ms can recognize and bind to specific tumor-associated antigens on tumor cells, leading to their engulfment and destruction. Such phagocytic activity helps to clear tumor cells and reduce tumor burden 48, 78, 80, 143.

**Cytokine Production:** Upon activation, CAR-Ms can produce and secrete a range of cytokines that modulate the immune response. For example, proinflammatory cytokines such as interleukin-12 (IL-12) and IFN-γ can promote antitumor immunity, which can be suppressed by anti-inflammatory cytokines such as IL-10 and transforming growth factor-beta (TGF-β) 48, 78, 80, 143.

**Activation of Anti-tumor Immunity:** CAR-Ms have been shown to play a key role in the immune response to BTs, not only by actively seeking out and destroying tumor cells through direct phagocytosis but also by recruiting and activating other immune cells to the tumor site. These immune cells, including T cells, NK cells and dendritic cells, assist in improving overall tumor clearance. Additionally, CAR-Ms can induce adaptive antitumor immunity by processing and presenting tumor antigens to T cells, leading to the activation of tumor-specific cytotoxic T lymphocytes (CTLs), which improves tumor clearance 78, 143.

**Reprogramming of the TME:** BTs are known for highly immunosuppressive microenvironment, which can hinder the effectiveness of immune-based therapies. CAR-Ms can help counteract such immunosuppression by producing factors that reprogram the microenvironment, converting it into a more immunostimulatory state, including reducing the amount of immunosuppressive cells such as myeloid-derived suppressor cells (MDSCs) and Tregs, as well as downregulating the expression of immune checkpoint molecules such as PD-L1 48, 78, 80.

With the immunomodulatory effect of CAR-Ms in the tumor microenvironment, CAR-Ms have potential to overcome the immunosuppressive barriers associated with these types of cancer and enhance the natural defense mechanisms of human body against tumor cells. However, further research and clinical trials are needed to optimize the design and delivery of CAR-Ms for BTs treatment as well as to fully understand their safety and efficacy profiles.

## STRATEGIES FOR OPTIMIZING CAR-M THERAPHY IN BRAIN TUMOR TREATMENT

### Target Selection

Identifying and validating the specific targets expressed in tumor cells will decrease off-target effects, increase specificity, leading to effective target selection for BTs treatment 144. Bioinformatics tools are capable of analyzing large datasets in order to identify potential tumor-specific antigens and to predict their immunogenicity. Single-cell RNA sequencing offers insights into the tumor microenvironment, elucidating heterogeneity and identifying distinctive cell populations that can be targeted. The multi-target strategy has the potential to enhance therapeutic efficacy by addressing the challenges of tumor heterogeneity and antigenic escape 145, 146. A combination therapy targeting IL13Rα2 and HER2 by bispecific CAR-T cells co-expressing IL13Rα2 and HER2 CAR molecules has been shown to have significant potential for the elimination of tumor cells 147. By combining these technologies, off-target effects can be minimized and the efficacy of CAR-M therapies can be improved.

### CAR Design

Optimization of CAR design will enhance binding affinity, specificity, and signaling capacity 148. Costimulatory domains may also be incorporated to improve the activation and effector function of CAR-Ms 149, 150. The design of first-generation CAR-Ms draws upon the established framework of CAR-T cells, which nevertheless requires further refinement and innovation to facilitate enhanced CAR-M therapy. The therapeutic efficacy of CAR-M can be markedly enhanced by incorporating the intracellular signaling structural domains of TLR4 or IFN-γ into the CAR framework 151. The discovery of new CAR domains or signal pathway is necessary for the development of novel CAR-M therapy. Artificial Intelligence (AI) algorithms, which can integrate multi-omics data to predict the most effective constructs and optimize CAR design, may advance CAR-M therapy.

### Combination Therapy

The optimal design of CAR-M therapy is intended to facilitate macrophages to phagocytose tumor cells in the maximum extent, with the objective of eradicating the tumor. Nevertheless, the capacity of macrophages to phagocytize is markedly constrained by the phagocytosis checkpoint and immune checkpoint 152, 153. Consequently, the combined use of immune checkpoint inhibitors and phagocytic checkpoint inhibitors greatly enhanced *in vivo* macrophage phagocytosis and suppressed tumor development 154, 155. In addition, macrophage has the potential to promote T cell activation and potential advantages in infiltrating to BTs, while T cells has limited infiltrating ability into the dense extracellular matrix of tumor 156. It was demonstrated that CAR-M and CAR-T cells exhibited a more powerful antitumor reaction compared to each treatment individually. The inflammatory factors secreted by CAR-T cells augment the cytotoxicity of CAR-Ms by inducing M1 polarization and increase the expression of costimulatory ligand, that may promote the fitness and activation of CAR-T cells in turn 61. Through these approaches, the effectiveness of CAR-M therapy can be improved, ultimately leading to improved patient outcomes.

### Personalized Therapies and Monitoring

CAR-M therapy can be adapted to the specific characteristics of an individual tumor by developing CARs that target antigens expressed on the tumor cells. Patient-specific CAR-M therapy can be designed based on the distinctive tumor molecular subtypes of each patient. Methods for the non-invasive monitoring of CAR-M therapy, such as imaging technique, can be used to assess treatment efficacy, detect relapse, and guide potential modifications to the therapy 157, 158. Imaging techniques can be used to track the distribution and activity of CAR-Ms *in vivo* 159. Regular monitoring of biomarkers can help assess the effectiveness of CAR-M therapy and detect any adverse effects early on 160. Monitoring the immune response can provide insights into the therapy impact on the immune system and help in managing potential side effects 161. Additionally, the role of AI and Machine Learning (ML) in predicting effectiveness and response could offer a novel perspective on advancing personalized medicine in BTs treatment.

These strategies have the potential to significantly improve patient outcomes, but further research is needed to ensure the safe and effective implementation in clinical practice. The development of new strategies to enhance the safety and efficacy of CAR-M therapy for BTs treatment is an important step towards improving patient outcomes. However, it is important to note that many of these strategies are still at the preclinical or early clinical stages of development. It is required to ensure in more research that these strategies can be used safely and effectively in clinical practice.

## THE CHALLENGES FACING CAR-M THERAPHY

### The Challenges of Administration and Biodistribution

Despite the considerable potential of CAR-M as a powerful cancer immunotherapy, numerous challenges must be addressed to achieve the desired outcomes. The most significant limitation is the number of cells that can be obtained 9, 25. CAR-Ms exhibit minimal proliferation after injection *in vivo*, which may impact the efficacy of the treatment 162. Currently, the predominant approach for CAR cell therapy is peripheral intravenous infusion 163. When CAR-Ms are administered intravenously at a restricted dose, the number of infiltrated macrophages in BTs is not the full extent of the injected 164. In contrast to CAR-T cells, CAR-Ms, despite the capacity for substantial immersion to the tumor microenvironment, potentially due to larger size or *in vivo* migratory characteristics, tend to accumulate in the lung, liver, and kidney, which may influence the effectiveness of treatment 48, 78. It was observed that the administration of CAR-Ms via peritoneal injection resulted in an enrichment of CAR-Ms in the intra-abdominal tumor tissue, implying that intertumoral or subarachnoid administration may be a better administration way 163.

### The Challenges of Clinical Translation

The production of CAR-M involves complex processes, including the isolation, genetic modification, and expansion of macrophages. The high cost and complexity of manufacturing remain the primary challenges to the clinical application of current CAR-based immunotherapies 165. Efforts to control costs are focused on the refinement of manufacturing techniques to improve the efficiency of the cell expansion, which could lead to a significant reduction in costs. It has been demonstrated that iPSC-derived macrophages facilitate the scalable production of therapeutic cells, which may result in a significant reduction in costs. Nevertheless, the technical challenges associated with the induction of functional and competent CAR-Ms from iPSCs remain significant 166. The *in vivo* editing technology can circumvent the extraction and expansion production cycle of CAR-Ms, representing a promising avenue of research and a crucial area of development for CAR-M therapy in the future 167. Several companies, including Carisma Therapeutics, are engaged in the development of CAR-M *in vivo* editing, but none have yet reached the clinical trial stage. Further research is required to enhance and standardize the manufacturing protocol for the production of clinical-grade products 168.

Furthermore, CAR-M therapy must undergo rigorous preclinical and clinical trials to obtain regulatory approval. This includes demonstrating safety, efficacy, and quality control in accordance with guidelines set by regulatory agencies. The regulatory path for CAR-T cells has set a precedent that emphasizes stringent evaluation of safety and efficacy. However, the patient safety and therapeutic outcomes may be different implications cause of multifunctional role in immune modulation 169. The long-term effects of CAR-Ms are particularly controversial, given the potential to profoundly alter the dynamics of the immune system 170. The tailored regulatory approaches are necessary, considering the unique biological behaviors of macrophages and their interaction with the TME. The challenge for regulators is to develop guidelines that adequately address these concerns 171.

## POTENTIAL FOCUS OF FUTURE DEVELOPMENTS IN CAR-M THERAPHY

Despite recent advancements, CAR-M therapy remains in infancy and requires further development to become the next generation of immunotherapy in clinical. *In situ* gene editing for CAR-Ms, mRNA-based CAR gene engineering and the combination with other immunotherapies will be the emerging strategy for the next generation of CAR-Ms, which have already been discussed extensively. Here, we examine two additional potential focus of CAR-M therapy in BTs treatment.

### Microglia in CAR-M Therapy

Microglia, arising from progenitor cells in the embryonic yolk sac, are resident phagocytes in the brain parenchyma with highly efficient phagocytic abilities. Microglia are involved in maintaining brain homeostasis, responding to injury, and modulating immune responses within the brain 172. In the context of BTs, microglia are often the dominant macrophage population and can significantly influence tumor progression and the immune landscape. The tumor environment confers microglia with an immunosuppressive phenotype with weaker phagocytic capacity, antigen presentation and T cell-stimulatory function, which supports the tumor growth 173. As with CAR-M therapy, CAR editing may potentially be able to reverse the tumor-promoting phenotype of microglia and exert an anti-tumor effect on BTs. Especially, multiple ways have been demonstrated to differentiate iPSCs into microglia 174. As the most abundant immune cell with intrinsic properties and functional population in the brain, the potential of microglia for use in CAR-M therapy in the treatment of BTs is considerable.

### The Role of Imaging in CAR-M Therapy

Leveraging imaging modalities within CAR-M therapy holds immense promise in enhancing our comprehension of therapy efficacy and limitations. As discussed above, CAR-M therapy has achieved remarkable success in the treatment of BTs, but its application still faces challenges. Leveraging imaging modalities within CAR-Ms therapy holds immense promise in enhancing our comprehension of therapy efficacy and limitations 157. Moreover, imaging facilitates the assessment of therapeutic responses and the identification of potential obstacles impeding CAR-M efficacy, such as tumor heterogeneity, immune evasion mechanisms, and off-target effects 158. By elucidating the interplay between CAR-Ms and the complex tumor milieu, imaging empowers clinicians and researchers to tailor interventions, mitigate risks, and maximize therapeutic outcomes. Incorporating advanced imaging technologies into CAR-M therapy not only enhances the ability to monitor treatment efficacy but also fosters a deeper understanding of the underlying biological mechanisms governing CAR-M function.

#### Cellular and Molecular Imaging *in vitro*

*In vitro* cellular and molecular imaging plays a pivotal role in elucidating the intricate interactions between Chimeric Antigen Receptor (CAR) cells and tumor cells, offering invaluable insights for the development of future immunotherapies. Utilizing fluorescent proteins and their derivative biosensors has emerged as a powerful approach in this pursuit.

Fluorescent proteins, along with live-cell staining dyes and fluorescent-conjugated antibody labeling, enable the visualization of both tumor cells and CAR-Ms in real-time, facilitating the assessment of CAR therapy's antitumor efficacy 175. By tracking the expression and spatial distribution of specific signaling molecules at the subcellular level, fluorescent proteins encoded by genes provide a means to decipher the determinants and regulatory factors governing CAR cell function 176.

These sophisticated imaging tools have proven instrumental in unraveling the molecular mechanisms underlying CAR cell activation upon encountering tumor cells. By visualizing dynamic cellular processes and signaling events, researchers could gain critical insights into the intricacies of CAR macrophage-tumor cell interactions, thereby informing the refinement of CAR therapy strategies.

#### Imaging *in vivo*

The efficacy of the CAR-M therapy relies on the successful trafficking of CAR-Ms to the designated targets within BTs. Leveraging imaging-based strategies enables real-time tracking and visualization of CAR-Ms, offering invaluable insights into treatment outcomes. Assessment of *in vivo* process through imaging holds the potential to predict the success or failure of the treatment 159. Utilizing Bioluminescence Imaging (BLI) as a foundation, tumor imaging can be augmented with various imaging modalities including Positron Emission Tomography (PET), Magnetic Resonance Imaging (MRI), or Photoacoustic Imaging. This amalgamation of techniques allows for precise tracking of CAR-M distribution, providing a comprehensive view of cell localization, functionality, and interactions within the BTs microenvironment. The integration of these diverse imaging methodologies allows researchers to delve into the intricate dynamics of immune responses, thereby refining therapeutic strategies for enhanced cancer treatment outcomes 158, 177. Furthermore, imaging serves a crucial role in diagnosing and monitoring toxicities or adverse effects associated with CAR-M therapy. In particular, imaging is instrumental in assessing the clinical implications of cytokine release syndrome (CRS) and immune effector cell-associated neurotoxicity syndrome (ICANS) toxicity subsequent to CAR therapy, which PET/CT playing a central role 178. Imaging-based approaches offer invaluable insights into the dynamics of CAR-M therapy, enabling real-time assessment of treatment efficacy, localization of CAR-Ms, and detection of potential adverse effects. By providing a deeper understanding of treatment mechanisms and outcomes, imaging contributes significantly to the refinement and optimization of CAR-M therapy for enhanced cancer treatment.

## OUTLOOK

The prospect for CAR-M therapy in the treatment of BTs is promising yet multifaceted. While preclinical studies have unveiled the potential of CAR-Ms in targeting tumor cells and eliciting robust anti-tumor immune responses, several challenges lie ahead on the path to clinical translation. Addressing the complexity of the tumor microenvironment, optimizing CAR-M design and engineering, navigating regulatory pathways, and conducting rigorous clinical trials are pivotal steps in advancing CAR-M therapy towards clinical application. Collaborative interdisciplinary approaches, leveraging expertise from diverse fields, will be instrumental in overcoming these challenges and refining therapeutic strategies.

As research endeavors persist in delving deeper into the complexities of CAR-M therapy, it becomes increasingly evident that interdisciplinary collaborations and rigorous clinical validation are indispensable. These collaborative efforts, drawing upon the collective expertise of researchers across various disciplines, are essential for unlocking the full potential of CAR-M therapy as a transformative modality in the field of neuro-oncology.

By fostering synergistic partnerships between immunologists, oncologists, bioengineers, and regulatory experts, interdisciplinary collaborations enable the convergence of diverse perspectives and methodologies. This interdisciplinary approach facilitates a comprehensive understanding of CAR-M therapy, from its molecular mechanisms to its clinical application, thereby accelerating its translation into effective treatments for BTs.

Furthermore, clinical validation plays a pivotal role in bridging the gap between preclinical promise and clinical reality. Rigorous clinical trials, guided by robust scientific evidence and conducted in collaboration with healthcare professionals and regulatory authorities, are essential for assessing the safety, efficacy, and long-term outcomes of CAR-M therapy in patients with BTs.

In conclusion, although formidable challenges persist, the collective efforts of researchers, clinicians, and stakeholders in the field of CAR-M therapy offer a beacon of hope for revolutionizing the treatment landscape of BTs. With continued dedication to research, innovation, and clinical validation, CAR-M therapy holds the promise of ushering in a new era of precision medicine, providing renewed hope and improved outcomes for patients confronting this devastating disease.

## Figures and Tables

**Table 1:** Advantages and Limitations of CAR-Ms Compared to CAR T-Cells

	CAR-T	CAR-M
Advantages	- Adequate number of circulating T cells	- Extensive infiltration in most solid tumors - Anti-tumor activity through phagocytosis, presentation of tumor antigen to Th1 cells and production of anti-inflammatory factors
Limitations	- Difficulty in infiltrating solid tumors - Immunosuppressive tumor microenvironment limits survival and persistence - Heterogeneity and loss of tumor antigens - CRS and OTOT toxicity	- Need to maintain M1 differentiation phenotype - CRS and OTOT toxicity

**Table 2:** Clinical Studies of CAR Macrophages

Product	Target	Indication	Type of CAR-M	Phase	Country
CT-0508	Her2	Her2 overexpressing solid tumors	PBMC-Mac	1	USA
MAC-001	Her2	Her2-positive advanced gastric cancer	PBMC-Mac	Early 1	China
CT-0525	Her2	Her2 overexpressing solid tumors	PBMC	1	USA
MCY-M11	Mesothelin	Ovary cancer and peritoneal mesothelioma	PBMC	1	USA
MT-101	CD5	CD5+T-cell lymphomas	Myeloid cell	1/2	USA

## References

[1] van den Bent MJ, Geurts M, French PJ, Smits M, Capper D, Bromberg JEC (2023). Primary brain tumours in adults. The Lancet.

[2] Rivera M, Norman S, Sehgal R, Juthani R (2021). Updates on Surgical Management and Advances for Brain Tumors. Curr Oncol Rep.

[3] Sharma P, Aaroe A, Liang J, Puduvalli VK (2023). Tumor microenvironment in glioblastoma: Current and emerging concepts. Neurooncol Adv.

[4] Noy R, Pollard JW (2014). Tumor-associated macrophages: from mechanisms to therapy. Immunity.

[5] Qian BZ, Pollard JW (2010). Macrophage diversity enhances tumor progression and metastasis. Cell.

[6] Toledo B, Zhu Chen L, Paniagua-Sancho M, Marchal JA, Perán M, Giovannetti E (2024). Deciphering the performance of macrophages in tumour microenvironment: a call for precision immunotherapy. J Hematol Oncol.

[7] Qiu Y, Chen T, Hu R, Zhu R, Li C, Ruan Y (2021). Next frontier in tumor immunotherapy: macrophage-mediated immune evasion. Biomark Res.

[8] Mantovani A, Marchesi F, Malesci A, Laghi L, Allavena P (2017). Tumor-associated macrophages as treatment targets in oncology. Nat Rev Clin Oncol.

[9] Li N, Geng S, Dong Z-z, Jin Y, Ying H, Li H-W (2024). A new era of cancer immunotherapy: combining revolutionary technologies for enhanced CAR-M therapy. Mol Cancer.

[10] Maalej KM, Merhi M, Inchakalody VP, Mestiri S, Alam M, Maccalli C (2023). CAR-cell therapy in the era of solid tumor treatment: current challenges and emerging therapeutic advances. Mol Cancer.

[11] Liu Q, Li J, Zheng H, Yang S, Hua Y, Huang N (2023). Adoptive cellular immunotherapy for solid neoplasms beyond CAR-T. Mol Cancer.

[12] Brynskikh AM, Zhao Y, Mosley RL, Li S, Boska MD, Klyachko NL (2010). Macrophage Delivery of Therapeutic Nanozymes in A Murine Model of Parkinson'S Disease. Nanomedicine.

[13] Muthana M, Giannoudis A, Scott SD, Fang H-Y, Coffelt SB, Morrow FJ (2011). Use of Macrophages to Target Therapeutic Adenovirus to Human Prostate Tumors. Cancer Res.

[14] Li X, Liu R, Su X, Pan Y, Han X, Shao C (2019). Harnessing tumor-associated macrophages as aids for cancer immunotherapy. Mol Cancer.

[15] Abdin SM, Paasch D, Lachmann N (2024). CAR macrophages on a fast track to solid tumor therapy. Nat Immunol.

[16] Liang Y, Xu Q, Gao Q (2023). Advancing CAR-based immunotherapies in solid tumors: CAR- macrophages and neutrophils. Front Immunol.

[17] Hadiloo K, Taremi S, Heidari M, Esmaeilzadeh A (2023). The CAR macrophage cells, a novel generation of chimeric antigen-based approach against solid tumors. Biomark Res.

[18] Sloas C, Gill S, Klichinsky M (2021). Engineered CAR-Macrophages as Adoptive Immunotherapies for Solid Tumors. Front Immunol.

[19] Li J, Chen P, Ma W (2024). The next frontier in immunotherapy: potential and challenges of CAR-macrophages. Exp Hematol Oncol.

[20] Louis DN, Perry A, Wesseling P, Brat DJ, Cree IA, Figarella-Branger D (2021). The 2021 WHO Classification of Tumors of the Central Nervous System: a summary. Neuro Oncol.

[21] Aldape K, Zadeh G, Mansouri S, Reifenberger G, von Deimling A (2015). Glioblastoma: pathology, molecular mechanisms and markers. Acta Neuropathol.

[22] Society NBT Astrocytoma. 2024. https://braintumor.org/brain-tumors/about-brain-tumors/brain-tumor-types/astrocytoma/.

[23] Nabors LB, Portnow J, Ammirati M, Baehring J, Brem H, Brown P (2015). Central Nervous System Cancers, Version 1.2015. J Natl Compr Canc Netw.

[24] Segura PP, Quintela NV, García MM, del Barco Berrón S, Sarrió RG, Gómez JG (2023). SEOM-GEINO clinical guidelines for high-grade gliomas of adulthood (2022). Clin Transl Oncol.

[25] Chen Y, Yu Z, Tan X, Jiang H, Xu Z, Fang Y (2021). CAR-macrophage: A new immunotherapy candidate against solid tumors. Biomed Pharmacother.

[26] Fares J, Petrosyan E, Salhab HA, Dmello C, Fares Y The Immunology of Brain Tumors. Cham: Springer International Publishing. p. 1-20.

[27] Chen Y, Fan W, Zhao Y, Liu M, Hu L, Zhang W (2024). Progress in the Regulation of Immune Cells in the Tumor Microenvironment by Bioactive Compounds of Traditional Chinese Medicine. Molecules.

[28] Zhou Y, Cheng L, Liu L, Li X (2023). NK cells are never alone: crosstalk and communication in tumour microenvironments. Mol Cancer.

[29] Schaaf MB, Garg AD, Agostinis P (2018). Defining the role of the tumor vasculature in antitumor immunity and immunotherapy. Cell Death Dis.

[30] Bikfalvi A, da Costa CA, Avril T, Barnier JV, Bauchet L, Brisson L (2023). Challenges in glioblastoma research: focus on the tumor microenvironment. Trends Cancer.

[31] Broekman ML, Maas SLN, Abels ER, Mempel TR, Krichevsky AM, Breakefield XO (2018). Multidimensional communication in the microenvirons of glioblastoma. Nat Rev Neurol.

[32] Zhu S, Yi M, Wu Y, Dong B, Wu K (2021). Roles of tumor-associated macrophages in tumor progression: implications on therapeutic strategies. Exp Hematol Oncol.

[33] Gutmann DH, Kettenmann H (2019). Microglia/Brain Macrophages as Central Drivers of Brain Tumor Pathobiology. Neuron.

[34] Hambardzumyan D, Gutmann DH, Kettenmann H (2016). The role of microglia and macrophages in glioma maintenance and progression. Nat Neurosci.

[35] Pan Y, Yu Y, Wang X, Zhang T (2020). Tumor-Associated Macrophages in Tumor Immunity. Front Immunol.

[36] Liu J, Geng X, Hou J, Wu G (2021). New insights into M1/M2 macrophages: key modulators in cancer progression. Cancer Cell Int.

[37] Wang N, Liang H, Zen K (2014). Molecular mechanisms that influence the macrophage m1-m2 polarization balance. Front Immunol.

[38] Tu S, Lin X, Qiu J, Zhou J, Wang H, Hu S (2021). Crosstalk Between Tumor-Associated Microglia/Macrophages and CD8-Positive T Cells Plays a Key Role in Glioblastoma. Front Immunol.

[39] Chaudhary R, Morris RJ, Steinson E (2021). The multifactorial roles of microglia and macrophages in the maintenance and progression of glioblastoma. J Neuroimmunol.

[40] Thiounn N, Pages F Fau - Mejean A, Mejean A Fau - Descotes J-L, Descotes Jl Fau - Fridman W-H, Fridman Wh Fau - Romet-Lemonne J-L, Romet-Lemonne JL (2002). Adoptive immunotherapy for superficial bladder cancer with autologous macrophage activated killer cells. J Urol.

[41] Andreesen R, Hennemann B, Krause SW (1998). Adoptive immunotherapy of cancer using monocyte-derived macrophages: rationale, current status, and perspectives. J Leukoc Biol.

[42] Davey AS, Chandler NJ, Elazar A, Weinstein JY, Nguyen JV, Trenker R (2022). De novo-designed transmembrane domains tune engineered receptor functions. eLife.

[43] Rafiq S, Hackett CS, Brentjens RJ (2020). Engineering strategies to overcome the current roadblocks in CAR T cell therapy. Nat Rev Clin Oncol.

[44] Mao R, Kong W, He Y (2022). The affinity of antigen-binding domain on the antitumor efficacy of CAR T cells: Moderate is better. Front Immunol.

[45] Guedan S, Calderon H, Posey AD, Maus MV (2019). Engineering and Design of Chimeric Antigen Receptors. Mol Ther Methods Clin Dev.

[46] Smith R, Shen R (2023). Complexities in comparing the impact of costimulatory domains on approved CD19 CAR functionality. J Transl Med.

[47] Wang S, Yang Y, Ma P, Zha Y, Zhang J, Lei A (2022). CAR-macrophage: An extensive immune enhancer to fight cancer. eBioMedicine.

[48] Klichinsky M, Ruella M, Shestova O, Lu XM, Best A, Zeeman M (2020). Human chimeric antigen receptor macrophages for cancer immunotherapy. Nat Biotechnol.

[49] Bu JY, Shaw AS, Chan AC (1995). Analysis of the interaction of ZAP-70 and syk protein-tyrosine kinases with the T-cell antigen receptor by plasmon resonance. Proc Natl Acad Sci U S A.

[50] Park SY, Kim IS (2017). Engulfment signals and the phagocytic machinery for apoptotic cell clearance. Exp Mol Med.

[51] Irving BA, Weiss A (1991). The cytoplasmic domain of the T cell receptor zeta chain is sufficient to couple to receptor-associated signal transduction pathways. Cell.

[52] Hege KM, Roberts MR (1996). T-cell gene therapy. Curr Opin Biotechnol.

[53] Braendstrup P, Levine BL, Ruella M (2020). The long road to the first FDA-approved gene therapy: chimeric antigen receptor T cells targeting CD19. Cytotherapy.

[54] Sadelain M, Riviere I, Brentjens R (2003). Targeting tumours with genetically enhanced T lymphocytes. Nat Rev Cancer.

[55] Asmamaw Dejenie T, Tiruneh GMM, Dessie Terefe G, Tadele Admasu F, Wale Tesega W, Chekol Abebe E (2022). Current updates on generations, approvals, and clinical trials of CAR T-cell therapy. Hum Vaccin Immunother.

[56] Xiao X, Huang S, Chen S, Wang Y, Sun Q, Xu X (2021). Mechanisms of cytokine release syndrome and neurotoxicity of CAR T-cell therapy and associated prevention and management strategies. J Exp Clin Cancer Res.

[57] Liu Q, Wu H, Li Y, Zhang R, Kleeff J, Zhang X (2020). Combined blockade of TGf-beta1 and GM-CSF improves chemotherapeutic effects for pancreatic cancer by modulating tumor microenvironment. Cancer Immunol Immunother.

[58] DeCordova S, Shastri A, Tsolaki AG, Yasmin H, Klein L, Singh SK (2020). Molecular Heterogeneity and Immunosuppressive Microenvironment in Glioblastoma. Front Immunol.

[59] Shimabukuro-Vornhagen A, Godel P, Subklewe M, Stemmler HJ, Schlosser HA, Schlaak M (2018). Cytokine release syndrome. J Immunother Cancer.

[60] D'Aloia MM, Zizzari IG, Sacchetti B, Pierelli L, Alimandi M (2018). CAR-T cells: the long and winding road to solid tumors. Cell Death Dis.

[61] Liu M, Liu J, Liang Z, Dai K, Gan J, Wang Q (2022). CAR-Macrophages and CAR-T Cells Synergistically Kill Tumor Cells In Vitro. Cells.

[62] Hu G, Guo M, Xu J, Wu F, Fan J, Huang Q (2019). Nanoparticles Targeting Macrophages as Potential Clinical Therapeutic Agents Against Cancer and Inflammation. Front Immunol.

[63] Hou T, Wang T, Mu W, Yang R, Liang S, Zhang Z (2020). Nanoparticle-Loaded Polarized-Macrophages for Enhanced Tumor Targeting and Cell-Chemotherapy. Nanomicro Lett.

[64] Gardell JL, Matsumoto LR, Chinn H, DeGolier KR, Kreuser SA, Prieskorn B (2020). Human macrophages engineered to secrete a bispecific T cell engager support antigen-dependent T cell responses to glioblastoma. J Immunother Cancer.

[65] Cha EB, Shin KK, Seo J, Oh DB (2020). Antibody-secreting macrophages generated using CpG-free plasmid eliminate tumor cells through antibody-dependent cellular phagocytosis. BMB Rep.

[66] Huang Y, Guan Z, Dai X, Shen Y, Wei Q, Ren L (2021). Engineered macrophages as near-infrared light activated drug vectors for chemo-photodynamic therapy of primary and bone metastatic breast cancer. Nat Commun.

[67] Pan K, Farrukh H, Chittepu V, Xu H, Pan CX, Zhu Z (2022). CAR race to cancer immunotherapy: from CAR T, CAR NK to CAR macrophage therapy. J Exp Clin Cancer Res.

[68] Bartok E, Hartmann G (2020). Immune Sensing Mechanisms that Discriminate Self from Altered Self and Foreign Nucleic Acids. Immunity.

[69] Nilsson M, Ljungberg J, Richter J, Kiefer T, Magnusson M, Lieber A (2004). Development of an adenoviral vector system with adenovirus serotype 35 tropism; efficient transient gene transfer into primary malignant hematopoietic cells. J Gene Med.

[70] Gaggar A, Shayakhmetov DM, Lieber A (2003). CD46 is a cellular receptor for group B adenoviruses. Nat Med.

[71] Laguette N, Sobhian B, Casartelli N, Ringeard M, Chable-Bessia C, Segeral E (2011). SAMHD1 is the dendritic- and myeloid-cell-specific HIV-1 restriction factor counteracted by Vpx. Nature.

[72] Lam E, Stein S, Falck-Pedersen E (2014). Adenovirus detection by the cGAS/STING/TBK1 DNA sensing cascade. J Virol.

[73] Engelman A (2005). The ups and downs of gene expression and retroviral DNA integration. Proc Natl Acad Sci U S A.

[74] Wang C, Pan C, Yong H, Wang F, Bo T, Zhao Y (2023). Emerging non-viral vectors for gene delivery. J Nanobiotechnology.

[75] Shin S, Lee P, Han J, Kim S-N, Lim J, Park D-H (2023). Nanoparticle-Based Chimeric Antigen Receptor Therapy for Cancer Immunotherapy. Tissue Eng Regen Med.

[76] Martinez FO, Gordon S (2014). The M1 and M2 paradigm of macrophage activation: time for reassessment. F1000Prime Rep.

[77] Mitra R, Fain-Thornton J, Craig NL (2008). piggyBac can bypass DNA synthesis during cut and paste transposition. EMBO J.

[78] Kang M, Lee SH, Kwon M, Byun J, Kim D, Kim C (2021). Nanocomplex-Mediated In Vivo Programming to Chimeric Antigen Receptor-M1 Macrophages for Cancer Therapy. Adv Mater.

[79] Latz E, Schoenemeyer A, Visintin A, Fitzgerald KA, Monks BG, Knetter CF (2004). TLR9 signals after translocating from the ER to CpG DNA in the lysosome. Nat Immunol.

[80] Chen C, Jing W, Chen Y, Wang G, Abdalla M, Gao L (2022). Intracavity generation of glioma stem cell-specific CAR macrophages primes locoregional immunity for postoperative glioblastoma therapy. Sci Transl Med.

[81] Dimitri A, Herbst F, Fraietta JA (2022). Engineering the next-generation of CAR T-cells with CRISPR-Cas9 gene editing. Mol Cancer.

[82] Jiang F, Doudna JA (2017). CRISPR-Cas9 Structures and Mechanisms. Annu Rev Biophys.

[83] Freund EC, Lock JY, Oh J, Maculins T, Delamarre L, Bohlen CJ (2020). Efficient gene knockout in primary human and murine myeloid cells by non-viral delivery of CRISPR-Cas9. J Exp Med.

[84] Luo YL, Xu CF, Li HJ, Cao ZT, Liu J, Wang JL (2018). Macrophage-Specific in Vivo Gene Editing Using Cationic Lipid-Assisted Polymeric Nanoparticles. ACS Nano.

[85] Ray M, Lee YW, Hardie J, Mout R, Yesilbag Tonga G, Farkas ME (2018). CRISPRed Macrophages for Cell-Based Cancer Immunotherapy. Bioconjug Chem.

[86] Willingham SB, Volkmer JP, Gentles AJ, Sahoo D, Dalerba P, Mitra SS (2012). The CD47-signal regulatory protein alpha (SIRPa) interaction is a therapeutic target for human solid tumors. Proc Natl Acad Sci U S A.

[87] Zhang J, Webster S, Duffin B, Bernstein MN, Steill J, Swanson S (2023). Generation of anti-GD2 CAR macrophages from human pluripotent stem cells for cancer immunotherapies. Stem Cell Reports.

[88] Xudong W, Siyu S, Yuqing Z, Xiaolong C, Chen C, Leilei C (2023). Metabolic Reprogramming via targeting ACOD1 promotes polarization and anti-tumor activity of human CAR-iMACs in solid tumors. Nat Commun.

[89] Huntley S, Baggott DM, Hamilton AT, Tran-Gyamfi M, Yang S, Kim J (2006). A comprehensive catalog of human KRAB-associated zinc finger genes: insights into the evolutionary history of a large family of transcriptional repressors. Genome Res.

[90] Gilbert LA, Larson MH, Morsut L, Liu Z, Brar GA, Torres SE (2013). CRISPR-mediated modular RNA-guided regulation of transcription in eukaryotes. Cell.

[91] Dong Y, Zhang S, Gao X, Yin D, Wang T, Li Z (2021). HIF1α epigenetically repressed macrophages via CRISPR/Cas9-EZH2 system for enhanced cancer immunotherapy. Bioact Mater.

[92] Su S, Lei A, Wang X, Lu H, Wang S, Yang Y (2022). Induced CAR-Macrophages as a Novel Therapeutic Cell Type for Cancer Immune Cell Therapies. Cells.

[93] Genin M, Clement F, Fattaccioli A, Raes M, Michiels C (2015). M1 and M2 macrophages derived from THP-1 cells differentially modulate the response of cancer cells to etoposide. BMC Cancer.

[94] Schildberger A, Rossmanith E, Eichhorn T, Strassl K, Weber V (2013). Monocytes, peripheral blood mononuclear cells, and THP-1 cells exhibit different cytokine expression patterns following stimulation with lipopolysaccharide. Mediators Inflamm.

[95] Themeli M, Kloss CC, Ciriello G, Fedorov VD, Perna F, Gonen M (2013). Generation of tumor-targeted human T lymphocytes from induced pluripotent stem cells for cancer therapy. Nat Biotechnol.

[96] Li Y, Hermanson DL, Moriarity BS, Kaufman DS (2018). Human iPSC-Derived Natural Killer Cells Engineered with Chimeric Antigen Receptors Enhance Anti-tumor Activity. Cell Stem Cell.

[97] Zhang L, Tian L, Dai X, Yu H, Wang J, Lei A (2020). Pluripotent stem cell-derived CAR-macrophage cells with antigen-dependent anti-cancer cell functions. J Hematol Oncol.

[98] Huo Y, Zhang H, Sa L, Zheng W, He Y, Lyu H (2023). M1 polarization enhances the antitumor activity of chimeric antigen receptor macrophages in solid tumors. J Transl Med.

[99] Lei A, Yu H, Lu S, Lu H, Ding X, Tan T (2023). A second-generation M1-polarized CAR macrophage with antitumor efficacy. Nat Immunol.

[100] Guo Q, Qian Z-M (2024). Macrophage based drug delivery: Key challenges and strategies. Bioact Mater.

[101] Hutchinson JA, Riquelme P, Sawitzki B, Tomiuk S, Miqueu P, Zuhayra M (2011). Cutting Edge: Immunological consequences and trafficking of human regulatory macrophages administered to renal transplant recipients. J Immunol.

[102] Reiss KA, Yuan Y, Ueno NT, Johnson ML, Gill S, Dees EC (2022). A phase 1, first-in-human (FIH) study of the anti-HER2 CAR macrophage CT-0508 in subjects with HER2 overexpressing solid tumors. J Clin Oncol.

[103] Annunziata CM, Ghobadi A, Pennella EJ, Vanas J, Powell C, Pavelova M (2020). Feasibility and preliminary safety and efficacy of first-in-human intraperitoneal delivery of MCY-M11, anti-human-mesothelin CAR mRNA transfected into peripheral blood mononuclear cells, for ovarian cancer and malignant peritoneal mesothelioma. J Clin Oncol.

[104] CellOrigin CellOrigin announced treatment of the first patient with CAR-M in China and reported the second generation of CAR-M for solid tumors. 2023. https://www.prnewswire.com/news-releases/cellorigin-announced-treatment-of-the-first-patient-with-car-m-in-china-and-reported-the-second-generation-of-car-m-for-solid-tumors-302020068.html.

[105] Mineo JF, Bordron A, Baroncini M, Maurage CA, Ramirez C, Siminski RM (2007). Low HER2-expressing glioblastomas are more often secondary to anaplastic transformation of low-grade glioma. J Neurooncol.

[106] Brennan CW, Verhaak RGW, McKenna A, Campos B, Noushmehr H, Salama SR (2013). The somatic genomic landscape of glioblastoma. Cell.

[107] Sepulveda-Sanchez JM, Vaz MA, Balana C, Gil-Gil M, Reynes G, Gallego O (2017). Phase II trial of dacomitinib, a pan-human EGFR tyrosine kinase inhibitor, in recurrent glioblastoma patients with EGFR amplification. Neuro Oncol.

[108] Byeon S, Hong JY, Lee J, Nam DH, Park SH, Park JO (2020). Use of Gefitinib in EGFR-Amplified Refractory Solid Tumors: An Open-Label, Single-Arm, Single-Center Prospective Pilot Study. Target Oncol.

[109] NCT02343406.

[110] Lassman AB, van den Bent MJ, Gan HK, Reardon DA, Kumthekar P, Butowski N (2019). Safety and efficacy of depatuxizumab mafodotin + temozolomide in patients with EGFR-amplified, recurrent glioblastoma: results from an international phase I multicenter trial. Neuro Oncol.

[111] Schuster J, Lai RK, Recht LD, Reardon DA, Paleologos NA, Groves MD (2015). A phase II, multicenter trial of rindopepimut (CDX-110) in newly diagnosed glioblastoma: the ACT III study. Neuro Oncol.

[112] O'Rourke DM, Nasrallah MP, Desai A, Melenhorst JJ, Mansfield K, Morrissette JJD (2017). A single dose of peripherally infused EGFRvIII-directed CAR T cells mediates antigen loss and induces adaptive resistance in patients with recurrent glioblastoma. Sci Transl Med.

[113] Johnson LA, Scholler J, Ohkuri T, Kosaka A, Patel PR, McGettigan SE (2015). Rational development and characterization of humanized anti-EGFR variant III chimeric antigen receptor T cells for glioblastoma. Sci Transl Med.

[114] Thaci B, Brown CE, Binello E, Werbaneth K, Sampath P, Sengupta S (2014). Significance of interleukin-13 receptor alpha 2-targeted glioblastoma therapy. Neuro Oncol.

[115] Jarboe JS, Johnson KR, Choi Y, Lonser RR, Park JK (2007). Expression of interleukin-13 receptor alpha2 in glioblastoma multiforme: implications for targeted therapies. Cancer Res.

[116] Debinski W, Gibo DM, Hulet SW, Connor JR, Gillespie GY (1999). Receptor for interleukin 13 is a marker and therapeutic target for human high-grade gliomas. Clin Cancer Res.

[117] Brown CE, Warden CD, Starr R, Deng X, Badie B, Yuan YC (2013). Glioma IL13Ralpha2 is associated with mesenchymal signature gene expression and poor patient prognosis. PLoS One.

[118] Brown CE, Hibbard JC, Alizadeh D, Blanchard MS, Natri HM, Wang D (2024). Locoregional delivery of IL-13Rα2-targeting CAR-T cells in recurrent high-grade glioma: a phase 1 trial. Nat Med.

[119] Brown CE, Alizadeh D, Starr R, Weng L, Wagner JR, Naranjo A (2016). Regression of Glioblastoma after Chimeric Antigen Receptor T-Cell Therapy. N Engl J Med.

[120] Chang K, Pastan I (1996). Molecular cloning of mesothelin, a differentiation antigen present on mesothelium, mesotheliomas, and ovarian cancers. Proc Natl Acad Sci U S A.

[121] Weidemann S, Gagelmann P, Gorbokon N, Lennartz M, Menz A, Luebke AM (2021). Mesothelin Expression in Human Tumors: A Tissue Microarray Study on 12,679 Tumors. Biomedicines.

[122] Tchou J, Wang L-C, Selven B, Zhang H, Conejo-Garcia J, Borghaei H (2012). Mesothelin, a novel immunotherapy target for triple negative breast cancer. Breast Cancer Res Treat.

[123] Zhang Q, Liu G, Liu J, Yang M, Fu J, Liu G (2021). The antitumor capacity of mesothelin-CAR-T cells in targeting solid tumors in mice. Mol Ther Oncolytics.

[124] Ling V, Wu PW, Spaulding V, Kieleczawa J, Luxenberg D, Carreno BM (2003). Duplication of primate and rodent B7-H3 immunoglobulin V- and C-like domains: divergent history of functional redundancy and exon loss. Genomics.

[125] Wang Z, Wang Z, Zhang C, Liu X, Li G, Liu S (2018). Genetic and clinical characterization of B7-H3 (CD276) expression and epigenetic regulation in diffuse brain glioma. Cancer Sci.

[126] Zhang C, Zhang Z, Li F, Shen Z, Qiao Y, Li L (2018). Large-scale analysis reveals the specific clinical and immune features of B7-H3 in glioma. Oncoimmunology.

[127] Zhang J, Wang J, Marzese DM, Wang X, Yang Z, Li C (2019). B7H3 regulates differentiation and serves as a potential biomarker and theranostic target for human glioblastoma. Lab Invest.

[128] Lemke D, Pfenning PN, Sahm F, Klein AC, Kempf T, Warnken U (2012). Costimulatory protein 4IgB7H3 drives the malignant phenotype of glioblastoma by mediating immune escape and invasiveness. Clin Cancer Res.

[129] Digregorio M, Coppieters N, Lombard A, Lumapat PN, Scholtes F, Rogister B (2021). The expression of B7-H3 isoforms in newly diagnosed glioblastoma and recurrence and their functional role. Acta Neuropathol Commun.

[130] Lin YJ, Mashouf LA, Lim M (2022). CAR T Cell Therapy in Primary Brain Tumors: Current Investigations and the Future. Front Immunol.

[131] Kato D, Yaguchi T, Iwata T, Katoh Y, Morii K, Tsubota K (2020). GPC1 specific CAR-T cells eradicate established solid tumor without adverse effects and synergize with anti-PD-1 Ab. Elife.

[132] Chen G, Wu H, Zhang L, Wei S (2020). High glypican-1 expression is a prognostic factor for predicting a poor clinical prognosis in patients with hepatocellular carcinoma. Oncol Lett.

[133] Lu H, Niu F, Liu F, Gao J, Sun Y, Zhao X (2017). Elevated glypican-1 expression is associated with an unfavorable prognosis in pancreatic ductal adenocarcinoma. Cancer Med.

[134] Harada E, Serada S, Fujimoto M, Takahashi Y, Takahashi T, Hara H (2017). Glypican-1 targeted antibody-based therapy induces preclinical antitumor activity against esophageal squamous cell carcinoma. Oncotarget.

[135] Su G, Meyer K, Nandini CD, Qiao D, Salamat S, Friedl A (2006). Glypican-1 is frequently overexpressed in human gliomas and enhances FGF-2 signaling in glioma cells. Am J Pathol.

[136] Wang S, Qiu Y, Bai B (2019). The Expression, Regulation, and Biomarker Potential of Glypican-1 in Cancer. Front Oncol.

[137] Gargett T, Ebert LM, Truong NTH, Kollis PM, Sedivakova K, Yu W (2022). GD2-targeting CAR-T cells enhanced by transgenic IL-15 expression are an effective and clinically feasible therapy for glioblastoma. J Immunother Cancer.

[138] Liu Z, Zhou J, Yang X, Liu Y, Zou C, Lv W (2023). Safety and antitumor activity of GD2-Specific 4SCAR-T cells in patients with glioblastoma. Mol Cancer.

[139] Mount CW, Majzner RG, Sundaresh S, Arnold EP, Kadapakkam M, Haile S (2018). Potent antitumor efficacy of anti-GD2 CAR T cells in H3-K27M(+) diffuse midline gliomas. Nat Med.

[140] Prapa M, Chiavelli C, Golinelli G, Grisendi G, Bestagno M, Di Tinco R (2021). GD2 CAR T cells against human glioblastoma. NPJ Precis Oncol.

[141] Masoumi J, Jafarzadeh A, Abdolalizadeh J, Khan H, Philippe J, Mirzaei H (2021). Cancer stem cell-targeted chimeric antigen receptor (CAR)-T cell therapy: Challenges and prospects. Acta Pharm Sin B.

[142] Tatari N, Maich WT, Salim SK, McKenna D, Venugopal C, Singh S (2020). Preclinical Testing of CAR T Cells in a Patient-Derived Xenograft Model of Glioblastoma. STAR Protoc.

[143] Niu Z, Chen G, Chang W, Sun P, Luo Z, Zhang H (2021). Chimeric antigen receptor-modified macrophages trigger systemic anti-tumour immunity. J Pathol.

[144] Moreira R, Nóbrega C, de Almeida LP, Mendonça L (2024). Brain-targeted drug delivery - nanovesicles directed to specific brain cells by brain-targeting ligands. J Nanobiotechnology.

[145] El-Sayes N, Vito A, Mossman K (2021). Tumor Heterogeneity: A Great Barrier in the Age of Cancer Immunotherapy. Cancers.

[146] Uslu U, June CH (2023). T-cell Therapies Targeting Multiple Cancer Antigens: The Power of Many. Cancer Immunol Res.

[147] Hegde M, Mukherjee M, Grada Z, Pignata A, Landi D, Navai SA (2016). Tandem CAR T cells targeting HER2 and IL13Rα2 mitigate tumor antigen escape. J Clin Invest.

[148] Roselli E, Frieling JS, Thorner K, Ramello MC, Lynch CC, Abate-Daga D (2019). CAR-T Engineering: Optimizing Signal Transduction and Effector Mechanisms. BioDrugs.

[149] Collins MA, Jung I-Y, Zhao Z, Apodaca K, Kong W, Lundh S (2022). Enhanced Costimulatory Signaling Improves CAR T-cell Effector Responses in CLL. Cancer Res Commun.

[150] Nguyen P, Okeke E, Clay M, Haydar D, Justice J, O'Reilly C (2020). Route of 41BB/41BBL Costimulation Determines Effector Function of B7-H3-CAR.CD28ζ T Cells. Mol Ther Oncolytics.

[151] Duan Z, Li Z, Wang Z, Chen C, Luo Y (2023). Chimeric antigen receptor macrophages activated through TLR4 or IFN-γ receptors suppress breast cancer growth by targeting VEGFR2. Cancer Immunol Immunother.

[152] Feng M, Jiang W, Kim BYS, Zhang CC, Fu Y-X, Weissman IL (2019). Phagocytosis checkpoints as new targets for cancer immunotherapy. Nat Rev Cancer.

[153] Preusser M, Lim M, Hafler DA, Reardon DA, Sampson JH (2015). Prospects of immune checkpoint modulators in the treatment of glioblastoma. Nat Rev Neurology.

[154] Gordon SR, Maute RL, Dulken BW, Hutter G, George BM, McCracken MN (2017). PD-1 expression by tumour-associated macrophages inhibits phagocytosis and tumour immunity. Nature.

[155] Sloas C, Gabbasov R, Anderson N, Abramson S, Klichinsky M, Ohtani Y (2021). 144 SIRPα deficient CAR-Macrophages exhibit enhanced anti-tumor function and bypass the CD47 immune checkpoint. Regular and Young Investigator Award Abstracts.

[156] Rosales C, Uribe-Querol E (2017). Phagocytosis: A Fundamental Process in Immunity. Biomed Res Int.

[157] Smith DA, Kikano E, Tirumani SH, de Lima M, Caimi P, Ramaiya NH (2022). Imaging-based Toxicity and Response Pattern Assessment Following CAR T-Cell Therapy. Radiology.

[158] Liu L, Yoon CW, Yuan Z, Guo T, Qu Y, He P (2023). Cellular and molecular imaging of CAR-T cell-based immunotherapy. Adv Drug Deliv Rev.

[159] Naderer C, Hauser F, Hochreiner A, Axmann M, Jacak J (2023). Live Cell Imaging and in vivo Cell Tracking in Tissues. In: Walter A, Slezak P, Mueller R, Kerckhofs G, Bajoghli B, editors. Bioimaging in Tissue Engineering and Regeneration: Advanced Microscopy and Preclinical Imaging. Cham: Springer International Publishing.

[160] Levstek L, Janžič L, Ihan A, Kopitar AN (2024). Biomarkers for prediction of CAR T therapy outcomes: current and future perspectives. Front Immunol.

[161] Stark HL, Wang HC, Kuburic J, Alzhrani A, Hester J, Issa F (2021). Immune Monitoring for Advanced Cell Therapy Trials in Transplantation: Which Assays and When?. Front Immunol.

[162] Lu J, Ma Y, Li Q, Xu Y, Xue Y, Xu S (2024). CAR Macrophages: a promising novel immunotherapy for solid tumors and beyond. Biomarker Research.

[163] Dong X, Fan J, Xie W, Wu X, Wei J, He Z (2023). Efficacy evaluation of chimeric antigen receptor-modified human peritoneal macrophages in the treatment of gastric cancer. Br J Cancer.

[164] Kim J, Bae J-S (2016). Tumor-Associated Macrophages and Neutrophils in Tumor Microenvironment. Mediators Inflamm.

[165] Hu D, Yang R, Wang G, Li H, Fan X, Liang G (2024). Emerging Strategies to Overcome Current CAR-T Therapy Dilemmas - Exosomes Derived from CAR-T Cells. Int J Nanomedicine.

[166] Xue D, Lu S, Zhang H, Zhang L, Dai Z, Kaufman DS (2023). Induced pluripotent stem cell-derived engineered T cells, natural killer cells, macrophages, and dendritic cells in immunotherapy. Trends Biotechnol.

[167] Zhang Y, Yang J, Zhang T, Gu H (2023). Emerging advances in nanobiomaterials-assisted chimeric antigen receptor (CAR)-macrophages for tumor immunotherapy. Front Bioeng Biotechnol.

[168] Balke-Want H, Keerthi V, Cadinanos-Garai A, Fowler C, Gkitsas N, Brown AK (2023). Non-viral chimeric antigen receptor (CAR) T cells going viral. Immunooncol Technol.

[169] Yan L, Li J, Zhang C (2023). The role of MSCs and CAR-MSCs in cellular immunotherapy. Cell Commun Signal.

[170] Mantovani A, Allavena P, Marchesi F, Garlanda C (2022). Macrophages as tools and targets in cancer therapy. Nat Rev Drug Discov.

[171] Wills CA, Drago D, Pietrusko RG (2023). Clinical holds for cell and gene therapy trials: Risks, impact, and lessons learned. Mol Ther Methods Clin Dev.

[172] Nobs SP, Kopf M (2021). Tissue-resident macrophages: guardians of organ homeostasis. Trends Immunol.

[173] Mirzaei R, Yong VW (2022). Microglia-T cell conversations in brain cancer progression. Trends Mol Med.

[174] Csatári J, Wiendl H, Pawlowski M (2024). Forward programming human pluripotent stem cells into microglia. Trends Cell Biol.

[175] Kamikawa T, Hashimoto A, Yamazaki N, Adachi J, Matsushima A, Kikuchi K (2024). Bioisostere-conjugated fluorescent probes for live-cell protein imaging without non-specific organelle accumulation. Chem Sci.

[176] Tahir M, Anwar S, Mian A, Muzaffar AW (2022). Deep localization of subcellular protein structures from fluorescence microscopy images. Neural Comput Appl.

[177] Shi C, Zhang Q, Yao Y, Zeng F, Du C, Nijiati S (2023). Targeting the activity of T cells by membrane surface redox regulation for cancer theranostics. Nat Nanotechnol.

[178] Breen WG, Young JR, Hathcock MA, Kowalchuk RO, Thorpe MP, Bansal R (2023). Metabolic PET/CT analysis of aggressive Non-Hodgkin lymphoma prior to Axicabtagene Ciloleucel CAR-T infusion: predictors of progressive disease, survival, and toxicity. Blood Cancer J.

